# Comparison of the efficiency and performance of two systems and three membranes for blood feeding mosquitoes

**DOI:** 10.1186/s13104-021-05799-y

**Published:** 2021-10-09

**Authors:** Fatoumata Seck, Aurélie Cailleau, Mawlouth Diallo, Ibrahima Dia

**Affiliations:** 1 Pôle de Zoologie Médicale, Institut Pasteur de Dakar, 36 Avenue Pasteur, BP 220, Dakar, Senegal; 2 Ecole Inter-Etats des Sciences et Médecine Vétérinaire, Dakar, Senegal

**Keywords:** *Anopheles coluzzii*, Membrane, Blood feeding, Exposure time

## Abstract

**Objective:**

Due to different concerns in using appropriate mosquito blood feeding techniques, this work focused on evaluating the effectiveness of two artificial mosquito feeding systems (Rutledge and Hemotek) and three different membranes (Parafilm, mouse and chicken skins). Female mosquitoes from an *An. coluzzii* strain aged between 2 and 5 days were exposed to blood with the two systems at time intervals (5, 10, 15, 20, 25 and 30 min) with blood used on the day of collection, the next day and 2 days after.

**Results:**

Our results showed that the Hemotek system gave better blood feeding rates than the Rutledge system. Among the three membranes, the blood feeding rates with chicken and mouse skins were higher than those provided by the Parafilm membrane. Likewise, blood stored 1 day after collection gave higher levels than blood used on the day of collection and 2 days after. Regardless of the system, the lowest blood feeding rates were observed at 5 min compared to the other exposure times.

## Introduction

Artificial blood feeding of mosquitoes is routinely used in vector competence studies and in the maintenance of colonies of hematophagous mosquito species, including those that transmit arboviruses and malaria. For the specific case of vector competence studies, it facilitates the infection of mosquitoes with various pathogens including parasites, avoids the use of animals and thus direct host feeding experiments and allows the control of the titers of infectious agents [6]. Several methods have been developed to artificially blood feed mosquitoes. Their principle is based on warming the blood in a glass cylinder whose underside is covered with a thin membrane through which mosquitoes feed. Various membranes have been used to feed mosquitoes. These are animal skins, plastic films or Parafilm®. The final objective of these methods is to achieve high feeding success in the shortest possible time to overcome the possible effect of viral titer decrease [3] or parasite exflagellation in the specific case of *Plasmodium* [2]. The objective of this study was to determine the success of mosquito blood-feeding using the system developed by Rutledge et al. [9] and the new Hemotek system recently developed by the Hemotek Company Ltd. (Great Harwood, UK) using three different membranes (chicken skin, mouse skin and Parafilm® membrane).

## Main text

### Methods

#### Mosquito colony

We used a strain of *An. coluzzii* colonized since 2008 from specimens collected in the outskirts of Essos in Yaounde [10] and reared in the insectary of the Medical Zoology Unit of Institut Pasteur in Dakar. It was maintained there under standard conditions at a temperature of 27 ± 2 °C, 75 ± 5% relative humidity and a photoperiod of 12:12 h. Larva were maintained in 30 cm × 21 cm × 6.5 cm trays at a density of about 300 first instar (L1) larva per tray with 1500 ml of deionised water. They were fed with a diet of finely grounded Novobel Flakes® (Animalis®, Paris, France). The pupae were collected daily and placed in small plastic cups which were introduced into adult emergence cages. Emerging adults were kept in collapsible 30 cm × 30 cm × 30 cm Lumite screen cages (BioQuip products, Rancho Dominguez, California, USA). They were fed with 10% (w/v) sucrose solution and females were blood-fed every 2 days on guinea pig and allowed to oviposit in Petri dish lined with a wet filter paper.

#### Experimental procedure

This study was part of a program that was ethically approved by the Comité National d’Ethique pour la Recherche en Santé (reference no. 125/MSAS/DPRS/CNERS, Dakar, Senegal, 15 September 2017).

The membranes tested were shaved mouse and depilated chicken skins and Parafilm® paper. The blood used in all the experiments came from a male sheep reared on the experimental farm of the Institut Pasteur Dakar in Mbao (14°43′46.11″ N, 17°19′30.78″ W). The blood samples were taken from the jugular vein of the animal, using a syringe, then the blood was collected directly into blood bags containing citrate phosphate dextrose-adenine (CPDA) as an anticoagulant. All experiments were performed in accordance with relevant guidelines and regulations. After collection, the blood bags were kept in a refrigerated cooler and then sent to the Medical Zoology Unit of the IPD in Dakar and stored at + 4 °C and the sheep released and kept on the farm.

Blood feeding experiments were run on D1 (the day of the blood sample), the following day (D2) and the following 2 days (D3).

Mosquito females aged between 2 and 5 days were used. A total of 30 mosquitoes were placed in cylindrical cardboard cups and were afforded to feed on a 10% sucrose solution. This latter was removed 24 h before blood feeding.

After starvation, female mosquitoes were exposed to blood meal using each membrane for 5, 10, 15, 20, 25 and 30 min. For each membrane, the experiment was repeated 3 times and carried out in parallel for the two systems.

#### Data analysis

For each system, membrane and exposure time, the blood feeding rates (BFR) were calculated as the number of blood fed mosquitoes out of the total number of mosquitoes exposed.

We evaluated the effect of the systems and membranes on the proportions of mosquitos’ blood-feeding using the Odds Ratio implemented on the package *fmsb* available from the R software version 3.3.1. [7].

### Results

During the three experiments, 3259 female mosquitoes were exposed to blood feeding during 6 replicates (2 for each of the 3 experiments). The BFR were significantly different between the two systems (49% for Hemotek and 42.3% for the Rudledge system, χ^2^ = 14.5, df = 1, p = 0.0001). The odds of blood feeding were higher for the Hemotek system (Table 1).

**Table 1:** Comparison of the blood feeding rates between the two systems and the three membranes

Parameters	Blood fed. no	OR	Lower CI	Upper CI	p value
Systems					
Hemotek	802 (49)	1			
Rutledge	686 (42.3)	0.73	0.63	0.85	< 0.001
Membranes					
Parafilm	220 (20.2)	1			
Chicken skin	648 (59.3)	6.24	5.13	7.61	< 0.001
Mouse skin	620 (57.3)	5.76	4.74	7.02	< 0.001
Experiments					
Experiment 1	461 (42.3)	1			
Experiment 2	594 (54.7)	1.83	1.52	2.21	< 0.001
Experiment 3	433 (40)	0.90	0.75	1.08	0.25
Time (in minutes)					
5	188 (34.5)	1			
10	289 (53.3)	2.54	1.95	3.32	< 0.001
15	255 (47)	1.84	1.42	2.41	< 0.001
20	224 (41.3)	1.41	1.08	1.84	0.01
25	264 (49)	2.02	1.56	2.64	< 0.001
30	268 (49)	2.01	1.54	2.62	< 0.001

OR: odds ratio; no: number; CI: Confidence interval; (): percentage

Of the 18 tests performed, respectively 21% (114/542), 63.3% (348/550) and 62.4% (340/545) of the females were fed with Parafilm® membrane, chicken skin and mouse skin while the respective levels were 19.5% (106/544), 55.4% (300/542) and 52.2% (280/536) for the Rutledge system. The OR from chicken and mouse skins provided similar levels of blood fed mosquitoes but the proportions were highly significant compared to the Parafilm® membrane (Table 1).

Between the three experiments, experiment 2 where mosquitoes were exposed to the blood used the day after it was collected, showed the higher proportion of blood fed mosquitoes than the first and the third experiment (OR = 1.83, 95% CI = 1.52–2.21, p < 0.001). No difference was observed between the first and the third experiment. Moreover, the study of feeding rates also showed a significant increase in feeding rates over time (Table 1).

### Discussion

To our knowledge, this is the first study that comparatively evaluate the effectiveness of two systems and three membranes to artificially feed mosquitoes. We used 2 to 5 days old *An. coluzzii* females from a laboratory colony in order to standardize the mosquito age and obtain better blood feeding rates as already observed by Robert [8]. Our results show that the Hemotek system was more efficient than the Rutledge system, possibly due to the speed of the blood meal warming. In fact, in the Hemotek system, the feeders are in stainless steel while for the Rutledge system, they are made of glass. The thermal conductivity of stainless steel is 15 times higher than that of glass [5] and therefore heats up the blood more quickly. In addition, several studies have shown that warming the blood meal in experiments with artificial exposure to mosquitoes increases the attraction and initiation of blood meal intake [4, 6, 9].

The blood feeding rates with chicken and mouse skins were similar but very different from those of Parafilm® membrane. This observation can be linked to the nature of the membranes. Behin [1], Rutledge et al. [9] and Novak et al. [6] observed that the use of membranes of animal origin gives higher rates of blood fed mosquitoes than those of plant or synthetic origin. This may be because the skins used are from potential mosquito hosts. Indeed, in a similar study carried out with other types of membrane, Behin [1] showed that chicken skin exhibited the higher blood feeding rates.

Regardless of the system considered, the experiment with the blood used the day after its collection gave highly significant blood feeding rates. Indeed, several studies have demonstrated the phagostimulating effect of ATP on the blood meal intake of mosquitoes mainly due to the presence of adenine and phosphate, which are also contained in the CPDA. Regarding the duration of the blood feeding time, the low blood feeding rates observed at 5 min whatever the system used, is a result of low exposure for mosquitoes which do not have sufficient time to feed completely. In addition, there would be a delay before mosquitoes start blood feed. Other possible explanations include the mean age of the selected female mosquitoes between 2 and 5 days, the youngest feeding more slowly than the others [8], a differential capacity of blood deprivation, the group effect for initiation of blood intake, some encouraging or inhibiting the blood feeding of others.

This study shows that while the Hemotek and Rutledge systems using Parafilm membrane, mouse and chicken skins are effective to blood fed mosquitoes, using blood stored 1 day after collection resulted in higher blood feeding rates than the blood from the day of the sample and 2 days after. The highest blood feeding rates are observed using chicken or mouse skins at time points higher than 5 min. Thus, the Hemotek system using either the mouse or the chicken skins, is more effective and could be recommended to achieve high feeding rates in the shortest possible time in the case of vector competence or colony maintenance studies.

## Limitations

This work was performed using a well-established *An. coluzzii* laboratory colony. It has shown whatever the systems and membranes considered that this colony feeds on the exposed blood. As mosquito feeding success might depend on the species, we would like to evaluate other mosquito species including vectors of malaria and arboviruses.

## Data Availability

All data generated or analyzed during this study are included in this manuscript.

## Abbreviations

BFR: Blood feeding rates
CI: Confidence interval
CPDA: Citrate phosphate dextrose-adenine
L1: First instar larva
OR: Odds ratio
w/v: Weight per volume
